# The Brain of Binge Drinkers at Rest: Alterations in Theta and Beta Oscillations in First-Year College Students with a Binge Drinking Pattern

**DOI:** 10.3389/fnbeh.2017.00168

**Published:** 2017-09-14

**Authors:** Eduardo López-Caneda, Fernando Cadaveira, Angeles Correas, Alberto Crego, Fernando Maestú, Socorro Rodríguez Holguín

**Affiliations:** ^1^Neuropsychophysiology Lab, Research Center in Psychology (CIPsi), School of Psychology, University of Minho Braga, Portugal; ^2^Department of Clinical Psychology and Psychobiology, University of Santiago de Compostela Santiago de Compostela, Spain; ^3^Laboratory of Cognitive and Computational Neuroscience, Center for Biomedical Technology Madrid, Spain; ^4^Department of Basic Psychology II, Complutense University of Madrid Madrid, Spain

**Keywords:** alcohol, binge drinking, adolescence, EEG, resting state, eLORETA

## Abstract

**Background:** Previous studies have reported anomalous resting brain activity in the electroencephalogram (EEG) of alcoholics, often reflected as increased power in the beta and theta frequency bands. The effects of binge drinking, the most common pattern of excessive alcohol consumption during adolescence and youth, on brain activity at rest is still poorly known. In this study, we sought to assess the pattern of resting-state EEG oscillations in college-aged binge drinkers (BDs).

**Methods:** Resting-state brain activity during eyes-open and eyes-closed conditions was recorded from 60 channels in 80 first-year undergraduate students (40 controls and 40 BDs). Cortical sources activity of EEG rhythms was estimated using exact Low-Resolution Electromagnetic Tomography (eLORETA) analysis.

**Results:** EEG-source localization analysis revealed that BDs showed, in comparison with controls, significantly higher intracranial current density in the beta frequency band over the right temporal lobe (parahippocampal and fusiform gyri) during eyes-open resting state as well as higher intracranial current density in the theta band over the bilateral occipital cortex (cuneus and lingual gyrus) during eyes-closed resting condition.

**Conclusions:** These findings are in line with previous results observing increased beta and/or theta power following chronic or heavy alcohol drinking in alcohol-dependent subjects and BDs. Increased tonic beta and theta oscillations are suggestive of an augmented cortical excitability and of potential difficulties in the information processing capacity in young BDs. Furthermore, enhanced EEG power in these frequency bands may respond to a neuromaturational delay as a result of excessive alcohol consumption during this critical brain developmental period.

## Introduction

According to the National Institute of Alcohol Abuse and Alcoholism, a *binge* is a pattern of drinking alcohol that brings blood alcohol concentration (BAC) to 0.08 g percent or higher, which corresponds to consuming five or more drinks for men and four or more for women within a 2-h interval (National Institute of Alcohol, Abuse and Alcoholism, 2004). This type of excessive alcohol use is a highly prevalent pattern, mostly among high school and college students (Courtney and Polich, 2009). Recent reports show that around one third of young Europeans and North Americans are binge drinkers (BDs; Kraus et al., 2016; SAMHSA, 2016).

This prevalent pattern has been related to an array of negative consequences, including traffic collisions, poor academic performance, risky sexual behavior and neurocognitive deficits (White and Hingson, 2013; Carbia and López-Caneda, 2016). Concerning the latter, studies using neuropsychological batteries have demonstrated that BDs exhibit poor performance on tasks involving a number of cognitive processes such as, verbal and prospective memory (Heffernan et al., 2010; Carbia et al., 2017), inhibitory control (Czapla et al., 2015) or decision making (Moreno et al., 2012).

Electrophysiological measures of brain activity have also proved to be efficient methods for the comprehension of neurocognitive function as well as for the understanding of dysfunctional processes linked to various psychiatric disorders, including alcoholism (Parvaz et al., 2011; Kamarajan and Porjesz, 2015). Impairments in a variety of neurophysiological parameters have been consistently observed in alcoholic patients both during task execution and during resting state conditions (Campanella et al., 2009). In the resting electroencephalogram (EEG)—i.e., the electrophysiological recording of oscillatory brain activity while the person is relaxed- increased power in the beta and theta frequency bands has been reported extensively. In this sense, findings from several laboratories have repeatedly showed that alcoholics manifest higher resting beta power than non-dependent subjects (Costa and Bauer, 1997; Rangaswamy et al., 2002, 2003; Coutin-Churchman and Moreno, 2008; Ehlers et al., 2010; Mumtaz et al., 2016; Meyers et al., 2017). Furthermore, increases in the beta band in relapsers in comparison with alcoholics who remained abstinent (Bauer, 2001; Saletu-Zyhlarz et al., 2004) and in non-alcoholic individuals compared to alcoholic relatives (Pollock et al., 1995; Rangaswamy et al., 2004), appears to strength the relationship between the alcoholic spectrum and the beta power.

As with beta rhythms (although perhaps less consistently), elevated theta activity at rest has been reported from resting-state EEG studies in alcohol abusers (Pollock et al., 1992; Rangaswamy et al., 2003; Mumtaz et al., 2016; but see Saletu-Zyhlarz et al., 2004; Coutin-Churchman et al., 2006 for different findings).

Despite the functional meaning of these higher beta and theta rhythms in alcoholics is still under discussion, the increased power in this frequency bands has often been interpreted as reflecting cortical hyperexcitability—for beta rhythms- and reduced-cognitive processing capacity—for theta frequencies (Rangaswamy et al., 2002, 2003; Porjesz and Begleiter, 2003; Campanella et al., 2009).

Although comparatively much less studied, electrophysiological measures of brain activity also appear to be sensitive to the BD pattern (López-Caneda et al., 2014a; Petit et al., 2014b). As such, anomalous brain responses have been documented in BD youths during cognitive tasks using event-related potentials (ERPs) and event-related oscillations (EROs; Crego et al., 2009; Maurage et al., 2012; Smith and Mattick, 2013; Petit et al., 2014a; Watson et al., 2014; López-Caneda et al., 2017). Despite its well documented potential for detecting neurofunctional anomalies in chronic alcoholics, brain activity during task-free resting states has been virtually unaddressed in the BD population. As such, to the best of our knowledge only two studies have directly assessed the effects of BD on the frequency spectrum of the whole brain at rest (Courtney and Polich, 2010; Correas et al., 2015). Specifically, the EEG study conducted by Courtney and Polich (2009) showed increased delta and beta power in high-BDs during passive viewing, which was considered a potential biomarker for future alcoholism. Correas et al. (2015), using magnetoencephalography (MEG), reported increased theta and decreased alpha power in the occipital region of young BDs during eyes-closed resting state, which was considered an initial sign of anomalous neural activity caused by BD in youth. Although both studies point to anomalies in neural activity at rest in young BDs, the neurofunctional effects of this pattern of binge alcohol intake on the resting brain are far from well-studied.

The current study is the first to use source localization analysis to examine multichannel EEG data to detect the active brain regions during eyes-open and eyes-closed resting state in young BDs. To this end, we used the exact Low Resolution Electromagnetic Tomography Analysis (eLORETA), a well-validated source reconstruction model for mapping source activations of scalp-recorded EEG (Pascual-Marqui, 2007; Pascual-Marqui et al., 2011). We aimed to identify potentially abnormal intracortical EEG patterns in young BDs as compared to age-matched controls in specific brain regions to provide new neurophysiological evidence that might prove useful for early detection of brain damage associated with the BD pattern. We hypothesized that young BDs will display anomalous patterns of brain activity compared to control subjects, specifically in those frequency spectra typically impaired in alcohol abusers, namely, theta, and beta frequencies.

## Materials and methods

### Participants

Eighty students from the Complutense University of Madrid (Spain) participated in the study. This study is framed within a research project aimed to assess brain damage associated with BD. In this project, neuropsychological, neurostructural and MEG measurements were also taken. Thus, the present study shares part of the sample with the recent MEG study from Correas et al. (2015). Participants were selected on the basis of their responses to a questionnaire that included the Spanish validated version of the Alcohol Use Disorder Identification Test (AUDIT; Guillamón et al., 1999), the question 10 of the Alcohol Use Questionnaire (“When you drink, how fast do you drink? 1 drink in 3 or more h; 1 drink in 2 h; Drinks per hour: 1, 2, 3, 4, 5, 6, 7, or more”; Townshend and Duka, 2002), and other information about alcohol and drugs consumption gathered through a semi-structured interview. According to NIAAA's BD definition, participants reaching BAC ≥ 0.08 g/dL at least once during the last month were classified as BDs. On the other hand, the control group consisted of students who have never reached an alcohol concentration of 0.08 g/dL. BAC was calculated based on the information of the drinking episodes of the last 6 months according to the following formula:

$$\begin{array}{l} BAC=\left(\frac{G}{W\times bw}\right)-mr\times DP \end{array}$$

where *G* corresponds to grams of alcohol consumed on one occasion (the occasion of greatest consumption in the last month); *W* is body weight (kilograms); *bw* is a constant related to the water content of the human body, with value 0.68 for males and 0.55 for females; *mr* is the metabolization rate with a value of 0.15 for males and 0.18 for females; and *DP* is the drinking period (hours). Consequently, 40 participants were classified as BDs (20 females) and 40 as controls (19 females).

Impulsivity was assessed by the Barratt Impulsiveness Scale (BIS-11; Patton et al., 1995) and psychopathological symptoms were measured by the Symptom Checklist-90 revised questionnaire (SCL-90-R; Derogatis, 1983). Exclusion criteria were: non-corrected sensory deficits, any episode of loss of consciousness for more than 20 min, history of traumatic brain injury or neurological disorder, personal history of psychopathological disorders (according to DSM-IV-TR criteria), family history of alcoholism or substance abuse in first degree relatives, consumption of medical drugs with psychoactive effects (e.g., sedatives or anxiolytics) during the week previous to the assessment, AUDIT scores ≥ 20, and use of illegal drugs except cannabis.

Participants were asked to refrain from consuming alcohol at least 24 h before the EEG session. They were submitted to a Breathalyzer test, and the assessment was only performed after verifying 0% breath alcohol level. Additionally, subjects were instructed not to smoke, or drink tea or coffee for at least 3 h before the assessment. All participants provided written informed consent prior to assessment. The study was approved by the Ethics Committee of the Complutense University of Madrid and the procedure was undertaken in accordance with the Code of Ethical Principles for Medical Research Involving Humans Subjects outlined in the Declaration of Helsinki.

### EEG acquisition

As part of a more extensive EEG study, 3 and 4 min of electroencephalographic signal were acquired during eyes-open and eyes-closed resting state, respectively. Each subject was seated in a comfortable armchair located in a light- and sound-attenuated electrically shielded room. The electroencephalogram (EEG) was recorded using a 64-channel ActiCap system (Brain Products, Munich, Germany). Electrodes were located on the scalp according to the 10/10 system. All active electrodes were referred to the nose tip and grounded with an electrode placed at Fpz. Vertical and horizontal electrooculogram activity was recorded to control for potentials evoked by eye movements and blinks. Electrode impedances were kept below 20 kΩ. EEG signals were continuously amplified and digitized at a rate of 500 Hz, and filtered on-line with a 0.01–100 Hz band pass filter.

### Data analysis

#### Demographic data

Demographic variables were analyzed by a Student's *t*-test or chi-square test for independent samples.

#### EEG processing

EEG data were processed with BrainVision Analyser software (Version 2.1). The signal was digitally filtered off-line with a 0.1–70 Hz band-pass filter and then corrected for ocular artifacts by the procedure developed by Gratton et al. (1983). It was then segmented into epochs of 4,000 ms for both eyes-open and eyes-closed resting state. Epochs exceeding ± 100 μV at any scalp electrode were rejected. The number of surviving epochs did not differ significantly between groups in the eyes-open condition (Control group: 42.2 ± 4.7; BD group: 42.2 ± 4.9) nor in the eyes-closed condition (Control group: 54.5 ± 6.6; BD group: 56.1 ± 5.4).

#### EEG-source localization analysis

For the estimation of cortical sources activity, EEG data from 60 channels were analyzed using eLORETA; free academic software available at http://www.uzh.ch/keyinst/loreta.htm (Pascual-Marqui et al., 2011). The eLORETA is a three-dimensional, discrete and linear weighted minimal norm inverse solution method. The weights endow the tomography with the property of exact localization to test point sources, yielding images of current density with exact localization (spatial resolution 5 mm; Pascual-Marqui, 2007, 2009). eLORETA images represent the electrical activity at each of the 6,239 voxels (spatial resolution 5 mm) in the neuroanatomic Montreal Neurological Institute (MNI) space (Mazziotta et al., 2001).

EEG data were re-referenced to the common average before fast Fourier transformation. Mean power for each of the four classical frequency bands was computed and averaged across epochs for each subject in eyes-open and eyes-closed conditions separately: delta (1–4 Hz), theta (4–8 Hz), alpha (8–12 Hz), and beta (12–30 Hz).

Voxel-by-voxel between-group comparisons of the spectral amplitude in source space were conducted using nonparametric statistical mapping method (SnPM; Nichols and Holmes, 2002) implemented in the eLORETA software package. For each single voxel, a *t*-test comparing amplitude estimates for groups at defined frequency bands was performed. Then, 5,000 randomizations with the SnPM procedure were applied to determine a statistical threshold (*p* < 0.05) corrected for multiple comparisons. Significant voxels were attributed to the corresponding Brodmann areas using the MNI space.

## Results

### Demographic results

Demographic and alcohol consumption data are summarized in Table 1. There were no significant differences between groups regarding age, handedness, regular use of cannabis (one or more times a week), general severity index (GSI) of the SCL-R, or BIS-11 scores. Groups differed significantly in age of onset of regular drinking, number of drinks in a standard day, number of drinks in a drinking episode, total AUDIT score, regular use of tobacco (one or more times a week) and BAC (*p* < 0.001 for all comparisons).

**Table 1:** Demographic and drinking characteristics of the control and binge drinking groups.

	**Controls**	**Binge drinkers**
*N* (females)	40 (19)	40 (20)
Age	18.13 ± 0.33	18.10 ± 0.30
Caucasian ethnicity (%)	100	100
Regular tobacco smokers	1	6^*^
Regular use of cannabis	0	0
Age of onset of regular drinking	16.66 ± 1.05	14.70 ± 1.15^*^
Number of drinks in a standard day	0.48 ± 0.90	4.26 ± 2.03^*^
Number of drinks in a drinking episode	0.95 ± 1.50	6.78 ± 4.70^*^
Blood alcohol concentration (BAC) in a drinking day (g/dL)	0.01 ± 0.02	0.17 ± 0.09^*^
BIS-11 total score	60.58 ± 8.26	63.77 ± 9.21
GSI score	0.31 ± 0.17	0.31 ± 0.15
Total AUDIT score	1.07 ± 1.56	7.42 ± 3.36^*^

* *p ≤ 0.05 significant differences between groups*.

### eLORETA results

**Figure 1:**
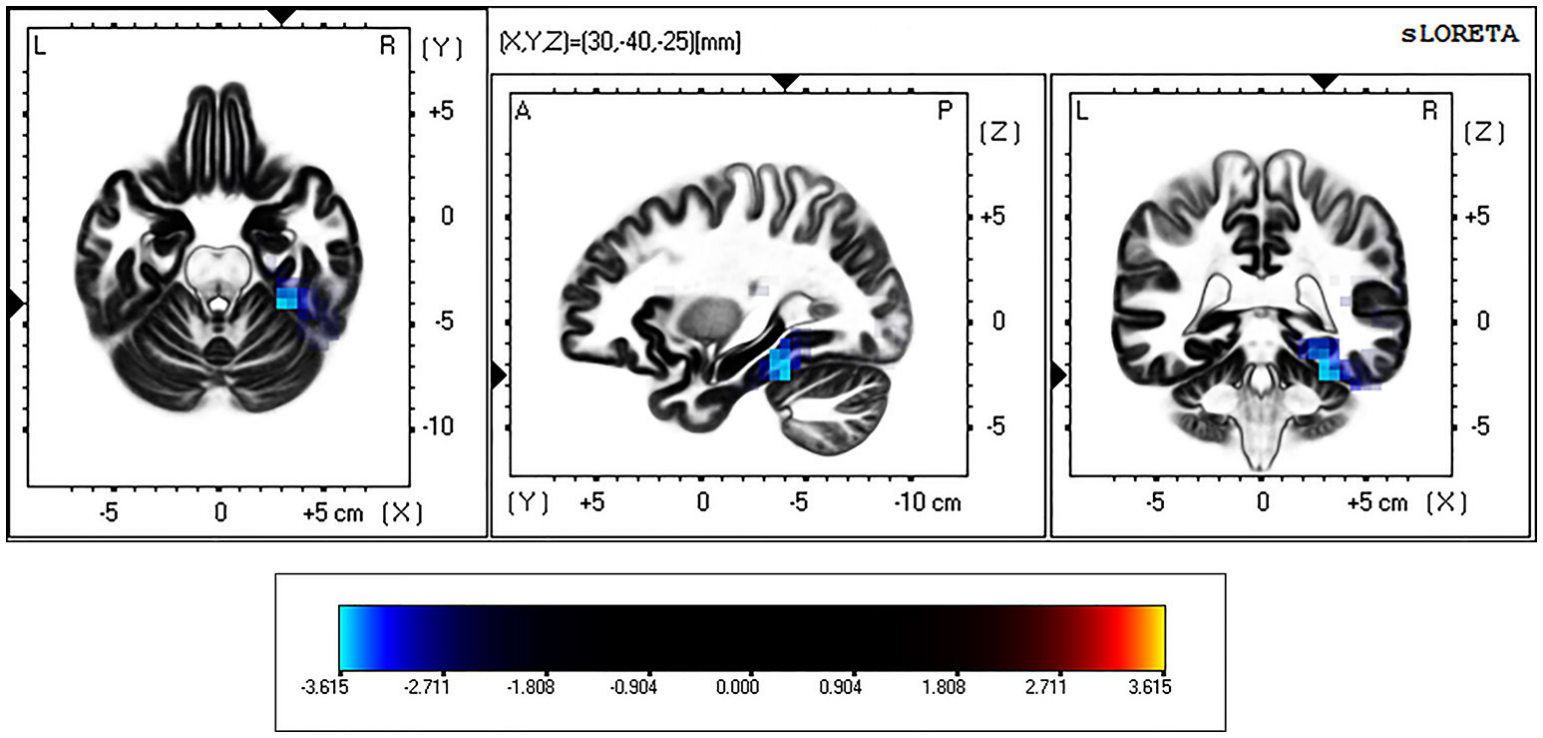
eLORETA-based statistical non-parametric maps (SnPM), comparing the exact current density values between control and binge drinking subjects during eyes-open resting state. Significantly higher current density (corrected *p* < 0.05) in the beta (12–30 Hz) frequency band in the binge drinking group relative to control group is shown in blue. L, Left; R, right; A, anterior; P, posterior.

eLORETA analysis revealed significant differences between controls and BDs at *p* < 0.05 after correction for multiple testing during eyes-open and eyes-closed conditions. Specifically, the current density in the beta frequency band during eyes-open resting state was significantly greater in the BD group in comparison with the Control group (*t* threshold required for significance: *t* = 3.115) in a large cluster involving the right parahippocampal and the fusiform gyri, as specified in Table 2 and illustrated in Figure 1.

**Table 2:** Summary of the brain areas with significantly higher current density in the beta frequency band during eyes-open resting state in the binge drinking group relative to the control group.

**Anatomical region**	**Brodmann area**	**MNI coordinates (*****x, y, z*****)**			*t*-score^*^
Temporal Lobe, Fusiform Gyrus	20	30,	−40,	−25	−3.56
		30,	−40,	−20	−3.54
		30,	−35,	−20	−3.53
		30,	−40,	−25	−3.52
		35,	−35,	−25	−3.46
		30,	−35,	−25	−3.44
		40,	−40,	−25	−3.26
	36	40,	−35,	−25	−3.22
		45,	−40,	−30	−3.16
	37	30,	−35,	−15	−3.50
		35,	−40,	−15	−3.25
		30,	−45,	−20	−3.23
		35,	−40,	−10	−3.13
		35,	−45,	−20	−3.12
Limbic Lobe, Parahippocampal Gyrus	27	25,	−35,	−5	−3.39
		25,	−30,	−10	−3.24
		25,	−30,	−5	−3.20
		20,	−35,	−5	−3.16
	35	20,	−35,	−10	−3.22
		20,	−35,	−15	−3.16
	36	25,	−35,	−15	−3.47
		25,	−40,	−15	−3.41
		25,	−35,	−20	−3.36
		25,	−40,	−10	−3.33
		35,	−35,	−15	−3.25
		30,	−30,	−20	−3.14
	37	30,	−40,	−15	−3.44
		30,	−40,	−10	−3.32

* *Corrected p < 0.05*.

**Figure 2:**
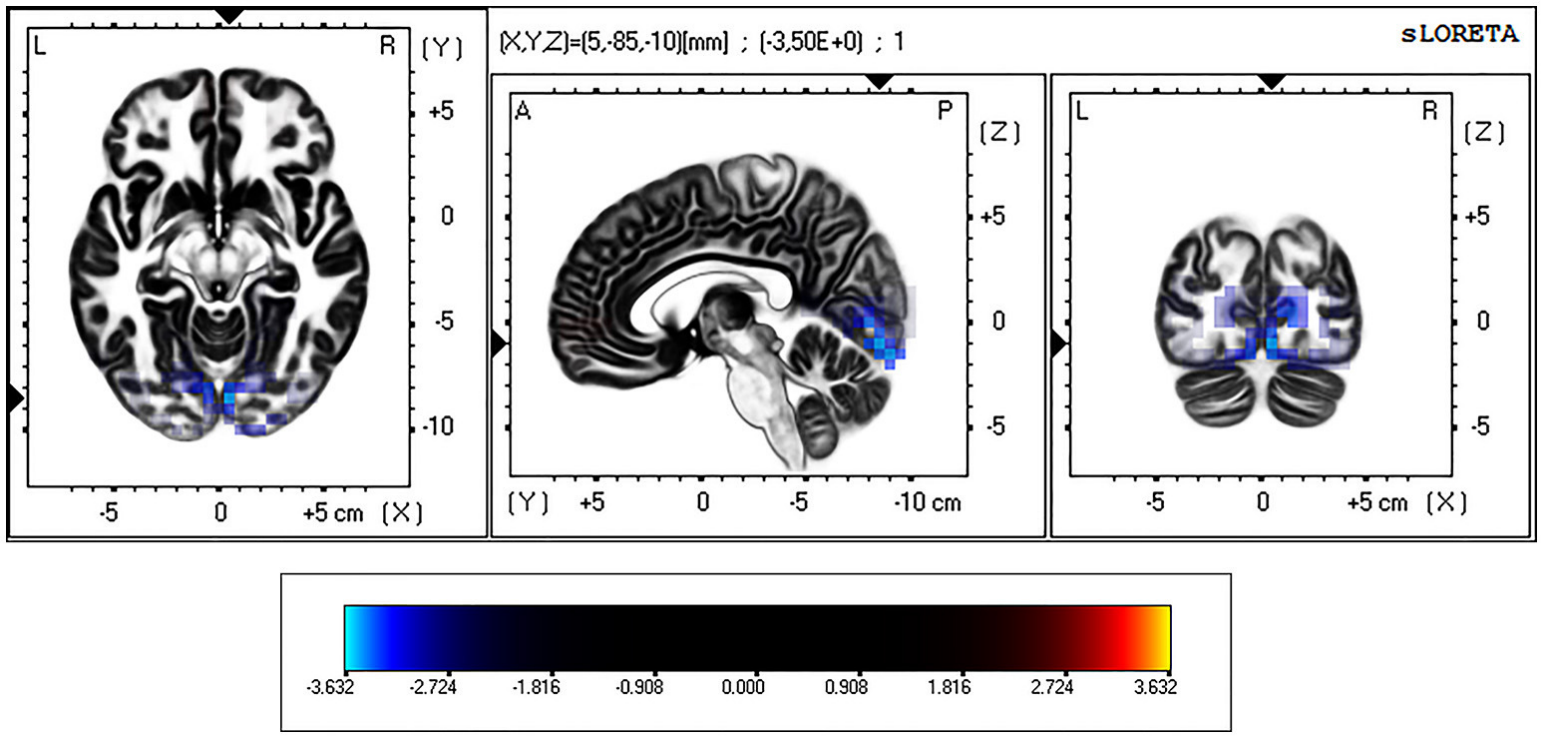
eLORETA-based statistical non-parametric maps (SnPM), comparing the exact current density values between control and binge drinking subjects during eyes-closed resting state. Significantly higher current density (corrected *p* < 0.05) in the theta (4–8 Hz) frequency band in the binge drinking group relative to control group is shown in blue. L, Left; R, right; A, anterior; P, posterior.

On the other hand, during the eyes-closed condition, the current density in the theta frequency band was significantly greater in the BD group in comparison with the Control group (*t* threshold required for significance: *t* = 3.194) in a large bilateral cluster involving the occipital cortex, specifically the cuneus and the lingual gyrus (see Table 3 and Figure 2).

**Table 3:** Summary of the brain areas with significantly higher current density in the theta frequency band during eyes-closed resting state in the binge drinking group relative to the control group.

**Anatomical region**	**Brodmann area**	**MNI coordinates (*****x, y, z*****)**			*t*-score^*^
Occipital Lobe, Cuneus	17	15,	−85,	5	−3.36
		10,	−85,	5	−3.32
		15,	−80,	10	−3.20
	18	15,	−85,	10	−3.28
Occipital Lobe, Lingual Gyrus	17	10,	−95,	−15	−3.37
		15,	−85,	0	3.35
		5,	−90,	−10	−3.28
		15,	−90,	0	−3.28
		15,	−95,	−15	−3.22
	18	5,	−85,	−10	−3.50
		5,	−90,	−15	−3.48
		5,	−80,	0	−3.40
		5,	−85,	−15	−3.39
		5,	−85,	−5	−3.37
		5,	−80,	−10	−3.36
		0,	−90,	−15	−3.36
		10,	−80,	−5	−3.34
		−5,	−85,	−15	−3.34
		−5,	−80,	−10	−3.34
		−5,	−90,	−20	−3.32
		5,	−95,	−15	−3.31
		5,	−90,	−20	−3.30
		−10,	−80,	−5	−3.29
		5,	−85,	−10	−3.29
		−5,	−95,	−20	−3.29
		10,	−85,	−15	−3.27
		−5,	−90,	−15	−3.26
		−10,	−80,	−10	−3.25
		−10,	−80,	0	−3.24
		5,	−80,	5	−3.24
		0,	−80,	0	−3.23
		10,	−80,	−10	−3.22
		−10,	−85,	−15	−3.22
		−15,	−80,	0,	−3.22

* *Corrected p < 0.05*.

## Discussion

**Figure 3:**
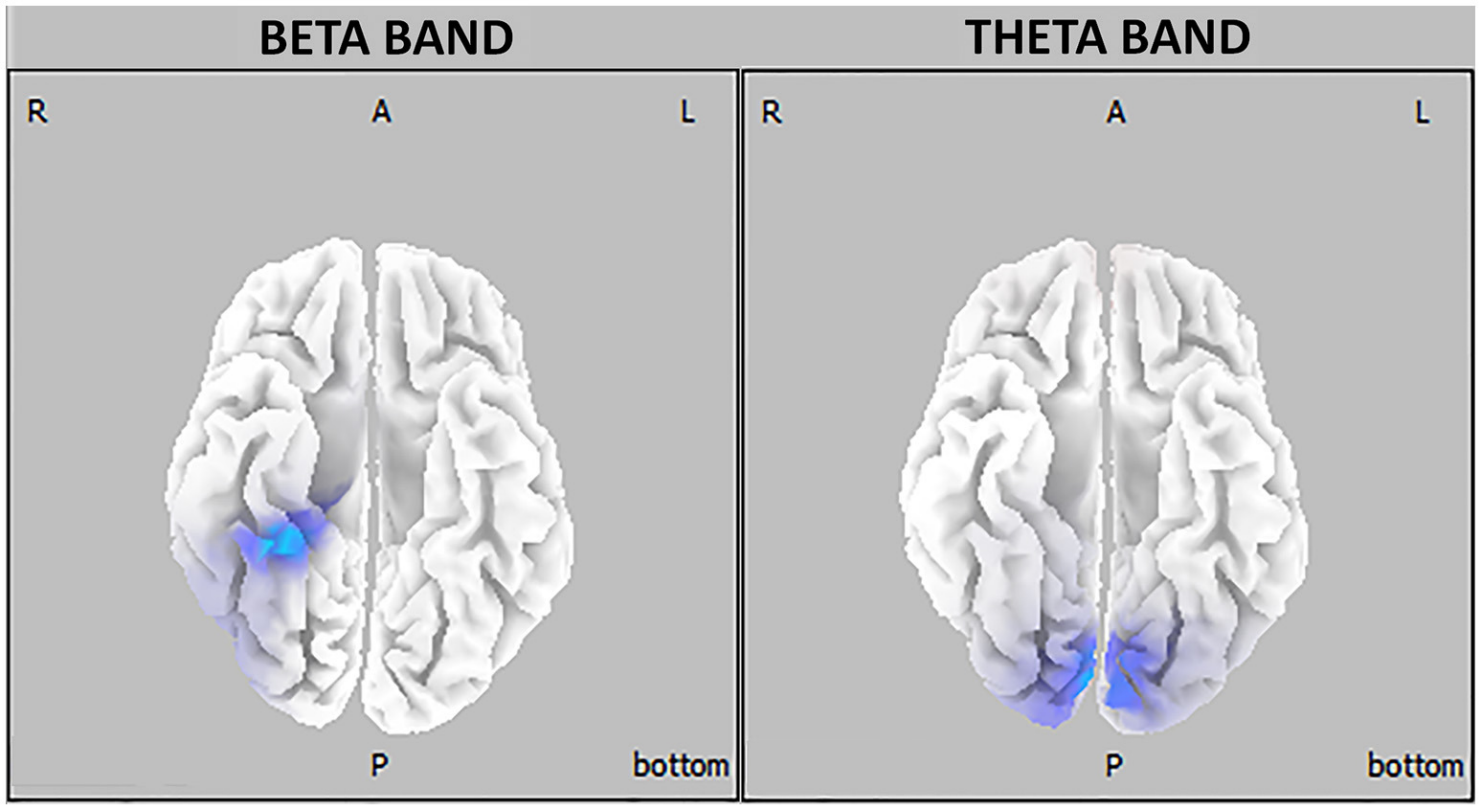
Three-dimensional eLORETA statistical maps of EEG oscillations showing between-groups differences in beta **(right)** and theta **(left)** frequency bands (blue-coded for *p* < 0.05, corrected for multiple testing).

The major finding of the present study is that young university students reporting a BD pattern displayed, in comparison with age-matched control subjects, a significantly higher intracranial current density in the beta frequency band in the right temporal lobe (parahippocampal and fusiform gyri) during eyes-open resting state as well as higher intracranial current density in the theta band in the bilateral occipital cortex (cuneus and lingual gyrus) during eyes-closed resting condition (see Figure 3).

As noted in the introduction, increased beta frequency rhythms in the EEG of alcoholics, mostly during rest, has been widely documented (Rangaswamy and Porjesz, 2014). Although it is difficult to associate oscillatory activity of a single frequency rhythm with a unique brain function, it has been proposed that tonic beta rhythms indicate states of enhanced arousal (Steriade et al., 1993; Engel and Fries, 2010). Thus, an imbalance in these oscillations may affect the overall level of neural excitation, so that an increment in the beta band may reflect a state of cortical hyperexcitability (Edenberg et al., 2004). In this sense, the increased beta power commonly observed in chronic alcoholics has been associated with a neural disinhibition resulting from an imbalance between excitatory and inhibitory neurons (Begleiter and Porjesz, 1999; Porjesz and Begleiter, 2003; Coutin-Churchman et al., 2006).

Our data extend the notion of abnormally increased cortical excitability seen in alcoholics to youths with a BD pattern, who also show higher beta power at rest than age-matched controls. In the same vein, the only previous study that, to our knowledge, has assessed the resting EEG of young BDs also observed increased beta- and delta-power in high-BDs during passive viewing (Courtney and Polich, 2010). According to these authors and to our results, this increment in beta power would be suggestive of brain overactivity in young BDs.

The results of the present study also revealed that the BD group exhibited higher theta power compared to the control group during eyes-closed resting state. As stated early, increased resting theta activity has been reported from several studies in alcohol abusers (Pollock et al., 1992; Rasgaswamy et al., 2003; Mumtaz et al., 2016).

In addition to alcoholics, enhanced tonic theta power has been observed in a variety of neurological disorders including Alzheimer's disease (Dauwels et al., 2010), vascular dementia (Babiloni et al., 2004), or attention deficit hyperactivity disorder (Burke and Edge, 2013). Likewise, increases in the tonic theta band have been remarked in altered neurophysiological states of the brain such as, drowsy and sleep states (Makeig et al., 2000; Tanaka et al., 2000) and after consumption of anesthetic drugs (Voss and Sleigh, 2007) or depressant substances such as, alcohol (Stenberg et al., 1994; Ehlers et al., 1998; Ilan and Gevins, 2001), which appears to suggest that when the ability to respond to external stimuli decreases, spontaneous theta activity increases (Klimesch, 1999).

It is important to note here that tonic activity differs from phasic or event-related changes in their origin and in their functional implications. While phasic changes are more or less under volitional control and take place at a rapid rate, tonic changes are (usually) not deliberate and occur over the lifetime in response to circadian rhythms, fatigue, distress, etc. (Klimesch, 1999). Regarding theta frequency band, tonic and phasic theta oscillations seem to be dissociated with respect to cognitive performance. In this sense, it has been reported that phasic theta power is enhanced as a function of task difficulty, memory load or attentional and cognitive control demands, and that increments in this phasic oscillation are positively associated with increasing cognitive performance (Klimesch et al., 2008; Nigbur et al., 2011; Cavanagh and Shackman, 2015; Clayton et al., 2015). Meanwhile, the opposite holds true for tonic theta activity, i.e., a pronounced suppression of tonic theta power is observed with increasing cognitive activity, whereas increments are associated with impaired global cognitive function (Jeong, 2004; Niedermeyer, 2005; Dubbelink et al., 2014). Consequently, large power in the range of tonic theta frequency has been considered an index of reduced cognitive processing capacity (Klimesch, 1999), and this is the reason why the higher resting theta activity present in the EEG of alcoholics has been suggested to reflect a deficit in information processing capacity (Rangaswamy et al., 2003; Campanella et al., 2009). Accordingly, the increased tonic theta power observed in the BD group of the present study might indicate a poorer information processing capacity compared to the control group.

Supporting the notion that BD may lead to similar spontaneous oscillatory activity as that identified in alcoholics, the only study examining whole brain activity measured by MEG in young BDs and conducted from our research group also reported higher theta power in the occipital region in BDs in comparison with controls during eyes-closed resting state (Correas et al., 2015). According to other BD studies assessing potential neural anomalies at the functional and structural level (Squeglia et al., 2012; Howell et al., 2013; Doallo et al., 2014; López-Caneda et al., 2014b; Correas et al., 2016), studies suggest that abnormal increases in the theta frequency band might correspond with a neuromaturational delay induced by a binge pattern of alcohol consumption during the adolescent period.

Adolescence is a key developmental stage with profound changes in brain morphology, generally reflected in an increase in global white matter volumes and a decrease in global gray matter structures (Lenroot and Giedd, 2006; Fuhrmann et al., 2015). Mirroring these neuromaturational changes, the oscillatory activity in the brain at rest is reduced in a broad frequency range from low-delta to high-beta between late childhood and early adulthood (Dustman et al., 1999; Boord et al., 2007; Lüchinger et al., 2012; Rodríguez-Martínez et al., 2017). Specifically, Whitford et al. (2007) and Buchmann et al. (2011) reported a reduction in gray matter volumes with age (ranging from 10 to 30 years and from 8 to 19 years, respectively) that was accompanied by a significant decrease in spectral power—most prominent in low frequencies—during awake and sleep EEG recordings. This juxtaposed decline in EEG power and gray matter has led to the suggestion that the synaptic pruning that typically takes place during the transition from childhood to adulthood is responsible for the observed reduction in EEG frequencies (Boord et al., 2007; Whitford et al., 2007; Feinberg and Campbell, 2010; Buchmann et al., 2011; Gómez et al., 2017).

Within the heterogeneous pattern of neurodevelopmental trajectories, the temporal cortex is the region where gray matter matures last, peaking generally around 17 years of age (Giedd, 2004; Gogtay et al., 2004), although some studies have extended this peak to around 30 years of age (Sowell et al., 2003). Taking into account the relationship between EEG power and gray matter changes, the increased beta power recorded over the right temporal cortex (parahippocampal and fusiform gyri) in the young BDs—aged 18 years—of our study might be related to neuroanatomical immaturity in these subjects compared to controls. As mentioned above, this would be in accordance with several neurostructural studies indicating enlarged gray matter (in some cases gender- and region-specific) in the BD population (Squeglia et al., 2012; Howell et al., 2013; Doallo et al., 2014; Kvamme et al., 2016; Morris et al., 2017). In particular, Kvamme et al. (2016) found greater gray matter volumes in female BDs in the bilateral fusiform gyrus and the right parahippocampal gyrus, showing that these regions may be affected—i.e., may undergo delayed synaptic pruning—due to BD. However, the lack of studies directly comparing electrophysiological and structural data in young BDs coupled with the fact that other studies have reported reduced instead increased cortical volumes in this population (Mashhoon et al., 2014; Squeglia et al., 2015) exhort us to be cautious in this interpretation.

Besides that, a key feature of EEG maturation is the progressive replacement of tonic theta power in the occipital region in favor of faster rhythms from childhood to adolescence and early adulthood, continuing until the third decade of life (Niedermeyer, 2005; Segalowitz et al., 2010). Indeed, despite resting theta activity having its maximum in occipital regions, this is not prevalent in normal adult waking EEG (Puligheddu et al., 2005; Kamarajan and Porjesz, 2015; Gómez et al., 2017). Thus, the higher occipital theta power observed in young BDs relative to age-matched controls may be linked to a slowing down in the reduction of theta rhythms that naturally occurs during development. However, further studies examining the development of theta activity in young BDs are warranted to confirm this hypothesis.

In summary, the present study used EEG-source localization analysis in order to identify potentially anomalous resting EEG frequency patterns in young BDs. Results showed a significantly higher intracranial current density in the beta frequency band over the right medial temporal cortex (parahippocampal and fusiform gyri) during eyes-open rest, and in the theta band over the bilateral occipital cortex (cuneus and lingual gyrus) during eyes-closed, in 18 year-old BDs compared to age-matched controls. These findings seem to be in line with previous results in alcohol-dependent subjects and BDs which observed increased beta and/or theta power following chronic or heavy alcohol drinking. As in these studies, the augmented tonic beta and theta oscillations obtained in our study suggest cortical hyperexcitability and potential difficulties in the information processing capacity of young BDs. The presence of this anomalous EEG pattern in young non-dependent BDs with a relatively short history of consumption could be due to more deleterious effects of alcohol on still-in-development brains, perhaps by means of a delay on neuromaturational processes. Finally, although additional research involving the study of spontaneous EEG rhythms in BDs is required, this abnormally elevated tonic neural activity might act as a potential marker of early brain damage associated with BD.

## Author contributions

EL and AnC collected the data. EL and AlC analyzed the data. SR, FC, and FM designed the study. EL wrote the first draft of the manuscript and all authors read, revised, and approved the final manuscript.

### Conflict of interest statement

The authors declare that the research was conducted in the absence of any commercial or financial relationships that could be construed as a potential conflict of interest.
